# "Soft and rigid" dithiols and Au nanoparticles grafting on plasma-treated polyethyleneterephthalate

**DOI:** 10.1186/1556-276X-6-607

**Published:** 2011-11-25

**Authors:** Václav Švorčík, Zdeňka Kolská, Ondřej Kvítek, Jakub Siegel, Alena Řezníčková, Pavel Řezanka, Kamil Záruba

**Affiliations:** 1 Department of Solid State Engineering, Institute of Chemical Technology, 16628 Prague, Czech Republic; 2 Department of Chemistry, J. E. Purkyně University, 40096 Ústí nad Labem, Czech Republic; 3 Department of Analytical Chemistry, Institute of Chemical Technology, 166 28 Prague, Czech Republic

**Keywords:** PET, plasma treatment, dithiols and gold nanoparticles grafting, XPS, FTIR, zeta potential, AFM

## Abstract

Surface of polyethyleneterephthalate (PET) was modified by plasma discharge and subsequently grafted with dithiols (1, 2-ethanedithiol (ED) or 4, 4'-biphenyldithiol) to create the thiol (-SH) groups on polymer surface. This "short" dithiols are expected to be fixed via one of -SH groups to radicals created by the plasma treatment on the PET surface. "Free" -SH groups are allowed to interact with Au nanoparticles. X-ray photoelectron spectroscopy (XPS), Fourier transform infrared spectroscopy (FTIR) and electrokinetic analysis (EA, zeta potential) were used for the characterization of surface chemistry of the modified PET. Surface morphology and roughness of the modified PET were studied by atomic force microscopy (AFM). The results from XPS, FTIR, EA and AFM show that the Au nanoparticles are grafted on the modified surface only in the case of biphenyldithiol pretreatment. The possible explanation is that the "flexible" molecule of ethanedithiol is bounded to the activated PET surface with both -SH groups. On the contrary, the "rigid" molecule of biphenyldithiol is bounded via only one -SH group to the modified PET surface and the second one remains "free" for the consecutive chemical reaction with Au nanoparticle. The gold nanoparticles are distributed relatively homogenously over the polymer surface.

## Introduction

The long-term research field of our scientific group is the modification of polymer surfaces, i.e. preparation of chemically active groups or species (e.g. radicals, conjugated double bonds, oxygen containing and other functional groups) on the polymer surface with the aim to increase the polymer surface "attractivity" for applications in tissue engineering and electronics [1-5].

There are several techniques, such as plasma discharge or irradiation with UV-light or ions, for modification of polymer surface [6,7]. A common feature of all these approaches is a degradation of the polymer macromolecule chains and often an increase in the nanoscale surface roughness. In our preliminary experiment, the polyethylene surface morfology was modified by Ar plasma discharge and subsequent etching of short molecular polymer fragments in water [6]. Another important phenomenon is a formation of free radicals and their subsequent reaction with oxygen from the ambient atmosphere. The newly formed oxygen-containing chemical functional groups render the material surface more wettable and increased wettability may facilitate the adsorption, e.g. cell adhesion receptors [7,8]. Another interesting property of radiation-modified polymers is the formation of conjugated double bonds between carbon atoms and increased electrical conductivity of the material which may support their colonization with living cells higher or adhesion of subsequently deposited metals [9,10].

The non-toxicity of gold is related to its well-known stability, non-reactivity and bioinertness. In addition, the gold can easily react with thiol (-SH) derivates giving Au-S bond formation. So that gold nanoparticles can be attached to the radicals, created on the polymer surface by plasma discharge or irradiation with UV-light or ions, by chemical reactions via -SH group [9-12].

In this work, the surface of the polyethyleneterephthalate (PET) was modified by plasma discharge and subsequently grafted with dithiol to introduce -SH groups. Dithiol is expected to be fixed via one of -SH groups to radicals created by the preceding plasma treatment on the polymer surface. The other "free" -SH group is alloved to interact with gold nanoparticle. The main goal of this study is to examine the effect of the plasma treatment and dithiol grafting on the binding of the gold nanoparticles to the polymer surface. Surface properties of the plasma-modified PET are studied by different experimental techniques: X-ray photoelectron spectroscopy (XPS), Fourier transform infrared spectroscopy (FTIR), electrokinetic analysis were used for the characterization of surface chemistry of the modified polymer and atomic force microscopy (AFM) for the study of surface morphology and roughness of treated polymers and "vizualization" of Au nanoparticles.

## Experimental

### Materials and polymer modification

The present experiments were performed on biaxially oriented PET (density 1.3 g cm^-3^, 50-μm foil, Goodfellow Ltd., Huntingdon, UK). PET was modified by Ar plasma in Balzers SCD 050 (Balzers Union AG, Darmstadt, Germany) at room temperature and under the following conditions: gas purity was 99.997%, flow rate 0.3 l s^-1^, pressure 10 Pa, electrode distance 50 mm, its area 48 cm^2^, chamber volume approximately 1, 000 cm^3^, plasma volume 240 cm^3^, discharge power 8.3 W, treatment time 180 s.

**Figure 1:**
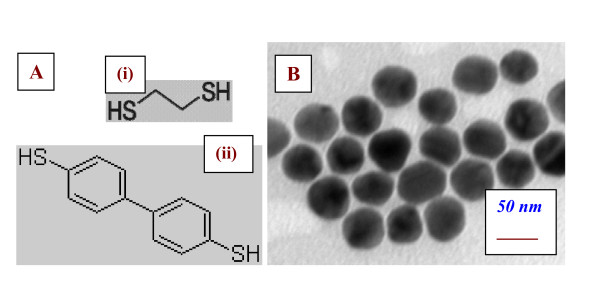
**Molecular structure and TEM images**. Molecular structure of (i) ethanedithiol (ED) and (ii) biphenyldithiol (BFD) (**A**); TEM images of Au nanoparticles from Transmission Electron Microscope (**B**). For structural characterization we used TEM (JEOL JEM-1010, Peabody, MA, USA) operated at 80 kV.

Immediately after the plasma treatment the samples were inserted into methanol solution of (1) 1, 2-ethanedithiol (ED) and (ii) 4, 4'-biphenyldithiol (BFD) (Figure 1A, 5.10^-3 ^mol l^-1^) for 2 h. In a control experiment, the etching of the polymer surface by methanol was also examined during 2-h exposure. Then the modified PET samples were immersed for 2 h into freshly prepared colloidal solution of Au nanoparticles (see Figure 1B), about 45 to 50 nm in diameter (citrate reduction preparation [13,14]). Finally, the samples were immersed in distilled water and dried with N_2 _flow.

### Diagnostic techniques

Properties of the PET samples-pristine or modified by the plasma treatment, by the etching and grafting with dithiol and Au nanoparticles were studied using various methods.

The changes of chemical structure were examined by FTIR on Bruker ISF 66/V spectrometer equipped with an Hyperion microscope with ATR (Ge) objective. The difference FTIR spectra, which are presented, were calculated as a difference of FTIR spectra measured on sample of PET plasma treated + etched in methanol and (1) plasma treated and grafted in solution of bihenyldithiol or (2) plasma treated + grafted in solution of biphenyldithiol + Au nanoparticles.

Electrokinetic analysis (zeta potential) of pristine and modified polymer samples was determined by SurPASS Instrument (Anton Paar, Austria). Samples were placed inside a cell with adjustable gap in contact with the electrolyte (0.001 mol dm^-3 ^KCl). For each measurement, a pair of samples with the same top layer was fixed on two sample holders (with a cross-section of 20 × 10 mm^2 ^and gap in between 100 μm) [15,16]. All samples were measured four times at a constant pH value with the relative error of 10%. For the determination of the zeta potential the streaming current and streaming potential methods were used and the Helmholtz-Smoluchowski and Fairbrother-Mastins equations were applied to calculate zeta potential [11,15,16].

Atomic contents of oxygen (1 s), carbon (1 s), sulphur (2 s) and gold (4f) in the surface layer of the modified polymer was determined from XPS spectra [17] recorded using an Omicron Nanotechnology ESCAProbeP spectrometer [18]. The results were evaluated using CasaXPS programme. Before the measurement, the samples were stored 2 weeks under standard laboratory conditions.

Surface morphology and roughness of pristine and modified PET were examined by AFM using VEECO CP II setup (both of tapping and phase modes). Si probe RTESPA-CP with the spring constant 0.9 N m^-1^. By repeated measurements of the same region (1 × 1 μm^2 ^in area), we certified that the surface morphology did not change after five consecutive scans. The mean roughness value (*R*_a_) represents the arithmetic average of the deviations from the centre plane of the sample.

## Results and discussion

### Chemical structure of plasma-modified and -grafted surface

Plasma treatment leads to cleavage of chemical bonds (C-H, C-C and C-O) [19]. The bond breaking leads to fragmentation of the polymer chain, to ablation of polymer surface layer and to creation of free radicals, conjugated double bonds and excessive oxygen containing groups [19]. Activated polymer surface can be grafted with thiol groups. The binding of the molecules is mediated by free radicals, present on the surface of the plasma-treated PET. The binding on new double bonds has not been proved [11]. Cleavage of the molecular chains facilitates solubility of the initially insoluble polymer in common solvents, e.g. water [9].

PET was modified in Ar plasma and then grafted from the methanol solution of ED or BFD and consecutively grafted with Au nanoparticles. Also a "blind" experiment was performed, where the interaction of methanol with plasma-treated PET was studied. The surface composition of PET (6-8 surface atomic layers) of pristine, plasma treated, dithiols grafted and coated with Au nanoparticles was investigated using XPS method. Atomic concentrations of C, O, S and Au in pristine and modified PET are shown in Table 1. From Table 1, it is evident that the surface of the pristine PET has dramatically lower oxygen concentration in comparison to theoretical value, the discrepancy being explained by re-orientation of surface polar groups value [17]. After the plasma treatment, the ogygen concentration increases due to formation of new oxygen groups on the chain sites where the bond cleavage of original polymeric chain occured [17]. It was shown previously that the carbonyl, carboxyl and ester groups are created on the polymer surface layers by the oxidation during or after the plasma treatment [20]. After the treatment with ED and BFD the concentration of oxygen in surface layer decreases. This can be explained by the "etching" of low-mass oxidized structures (LMWOS) [21]. After the treatment with ED and BFD, the XPS analysis revealed the presence of sulphur on the PET surface. The grafting with gold nanoparticles results in another decrease in the oxygen concentration and a decrease in the sulphur concentration as well. The decrease can be explained by consecutive etching of the plasma-treated surface layer in Au nanoparticles solution. The presence of gold was detected only in the case of PET grafted with biphenyldithiol. The pretreatment with ethanedithiol is not suitable for grafting with gold nanoparticles.

**Table 1:** Atomic concentrations of C (1*s*), O (1*s*), S (2*s*) and Au (4*f*)

Sample	Atomic concentrations of elements in at. %			
	Oxygen	Carbon	Sulphur	Gold
PET (theory)	28.6	71.4	-	-
Pristine PET [17]	2.4	91.6	-	-
PET/plasma	37.8	62.2	-	-
PET/plasma/ED	34.9	63.1	1.2	-
PET/plasma/BFD	31.5	67.1	1.4	-
PET/plasma/ED/Au	20.3	79.0	0.7	-
PET/plasma/BFD/Au	22.3	76.7	0.7	0.3

Atomic concentrations of C (1*s*), O (1*s*), S (2*s*) and Au (4*f*) in pristine PET (theory and present experiment [17]), plasma-treated sample (sample was measured 170 h after the plasma treatment), plasma treated + grafted in solution of (1) 1, 2-ethanedithiol (ED) or (2) biphenyl-4, 4'-dithiol (BFD) and (3) PET plasma treated + then grafted in ED or BFD + in gold nanoparticles respectively.

**Figure 2:**
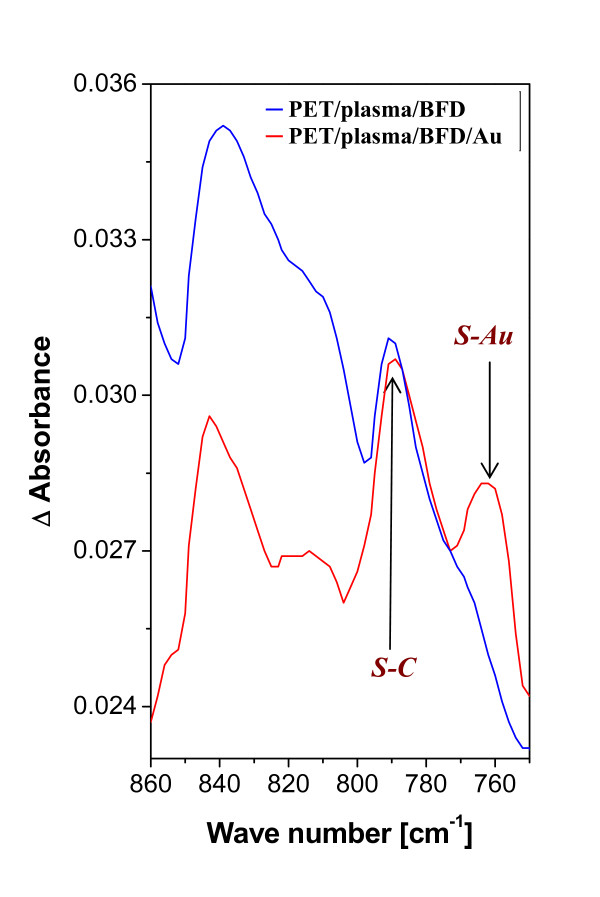
**Differential FTIR spectra**. (i) plasma treated and with biphenyldithiol grafted (PET/plasma/BFD) and (ii) plasma treated, grafted with BFD + then with Au nanoparticles (PET/plasma/BFD/Au).

FTIR spectroscopy was used for the characterization of chemical composition of modified PET samples. In Figure 2 the differential FTIR spectra of the PET samples (1) plasma treated and grafted in BFD and (2) treated and grafted with BFD and then with Au nanoparticles are shown. The band at 790 cm^-1 ^corresponds to absorption of the S-C group and the band at 761 cm^-1 ^is assigned to the S-Au group. After the grafting of plasma-treated PET with ethanedithiol and Au nanoparticles, the peak at 761 cm^-1 ^(S-Au) in FTIR spectra was not detected. This finding supports the conclusion that no Au nanoparticles are bonded to the PET treated in ethanedithiol.

From the results presented in Table 1 and Figure 2, it is apparent that Au nanoparticles are grafted only on the PET surface previously activated by biphenyldithiol. This can be explained by the concept that "flexible" molecule of ethanedithiol is bonded to activated polymer surface by both of -SH groups, while the more "rigid" molecule of biphenyldithiol is grafted only via one of -SH groups and the second one is "free" for chemical reaction with Au nanoparticle.

**Figure 3:**
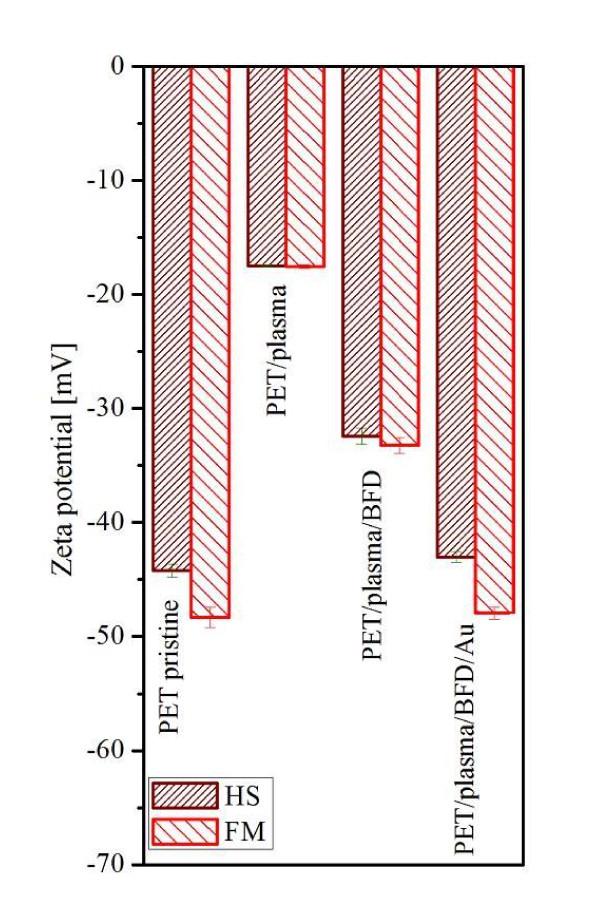
**Zeta potencial determined by SurPASS**. Pristine PET, plasma treated (PET/plasma), plasma treated + grafted with biphenyl-4, 4'-dithiol (PET/plasma/BFD) and plasma treated + grafted with BFD + then with Au nanoparticles (PET/plasma/BFD/Au).

**Figure 4:**
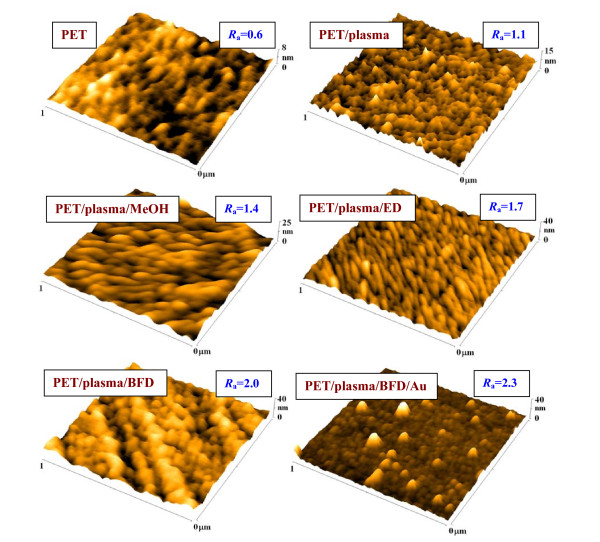
**AFM images of pristine PET, PET treated by plasma (PET/plasma), plasma treated and etched**. In (i) methanol (PET/plasma/MeOH), (ii) solution of ethanedithiol (PET/plasma/ED) and (iii) biphenyldithiol (PET/plasma/BFD), plasma treated + grafted with BFD + Au nanoparticles (PET/plasma/BFD/Au). *R*_a _is average surface roughness in nm.

Chemical structure of the modified PET films is expected to influence substantially their elektrokinetic potential in comparison with pristine PET. Zeta potentials (*ζ-*potential) for pristine PET, plasma-treated PET, plasma treated + grafted with BFD and plasma treated + grafted with BFD + with Au nanoparticles are presented in Figure 3. Zeta potential is affected by several factors, such as surface morphology, chemical composition (e.g. polarity, wetability) and electrical conductivity of surface. In our previous study [17], we found that in pristine PET the most of oxygen containing molecular segments are oriented towards the polymer bulk and the first atomic layers are effectively depleted of oxygen. This observation is supported also by the present data of Table 1. Plasma treatment results in a dramatic increase of the *ζ-*potential due to an increase in the concentration of more polar groups on the PET surface and corresponding increase of surface wetability. From Figure 3 it is evident, that BFD grafting leads to a dramatic decrease of the *ζ-*potential. This can be caused by the introduction of new groups (-SH) on the sample surface and by particular etching of surface-modified layer with BFD solution (i.e. change of sample's surface morphology, see AFM-Figure 4). Thiol groups in water surrounding dissociate a proton from these thiol groups, which leaves the surface with a negative charge. And zeta potential has the same sign as the surface charge. Due to this, the decrease of zeta potential confirms also the bonding of thiol groups on polymer surface. Another considerable decrease of the *ζ-*potential is apparent after the gold grafting procedure, which is due to the presence of electrically conductive Au nanoparticles.

### Surface morphology and homogeneity of Au nanoparticles on the modified PET

Surface morphology of pristine and modified PET was studied by AFM method. AFM images of pristine PET, PET-treated by plasma, plasma treated + etched in (1) methanol, (2) solution of ED and (3) BFD, plasma treated and grafted with BFD + Au nanoparticles are shown in Figure 4. The different scales of individual images were chosen to emphasize the changes in the surface morphology. From Figure 4, it is evident that the modification of PET by above-mentioned procedures has no significant effect on its surface roughness *R*_a_. The *R*_a _value "slightly" increases after the plasma treatment, surface etching and grafting with ED, BFD and gold nanoparticles. However the changes in the PET surface morphology are clearly visible. The change in surface morphology after the plasma treatment can be explained by preferential ablation of PET amorphous part of polymer. [19]. It can be asssumed, that the low-mass oxidized structures are preferentially dissolved in methanol and in ED and BFD solutions [21]. More significant change in the surface morphology after gold nanoparticles grafting is apparent. The "pyramidal" structures, relatively "homogeneously" spread on the polymer surface, can be due to the presence of the gold nanoparticles. Their "non-globular" shape in probably caused with the convolution of the tip with the sample's surface.

**Figure 5:**
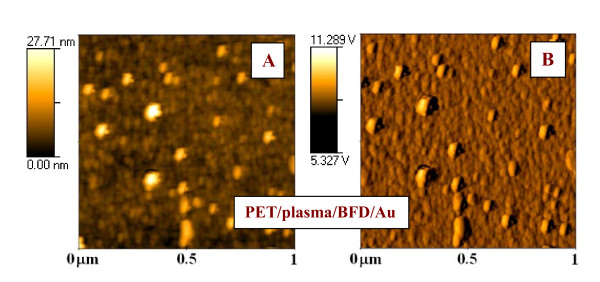
**AFM images of plasma-treated PET, grafted by biphenyldithiol + then grafted with Au nanoparticles**. Taken in tapping (**A**) and phase mode (**B**).

For the sake of clarity, the 2D AFM images of PET treated by plasma, grafted by BFD and then with gold nanoparticles, taken in tapping and phase mode, and are presented in Figure 5. It is obvious that the gold nanoparticles are spread relatively homogeneously on the polymer surface. At some randomly distributed places the aggregation of individual gold nanoparticles takes place. Gold nanoparticles do not create continuous coverage of the polymer surface and it is therefore not surprising that the electrical conductance remains unchanged in comparison with pristine polymers [11].

The gold nanoparticles homogenously distributed over the polymer surface could have a positive effect on the interaction with living cells, the effect which could be interesting for tissue engineering [9] The presence of gold nanoparticles may also facilitate adhesion of other gold structures to polymeric substrates, which can be useful for electronics [11].

## Conclusion

**Figure 6:**
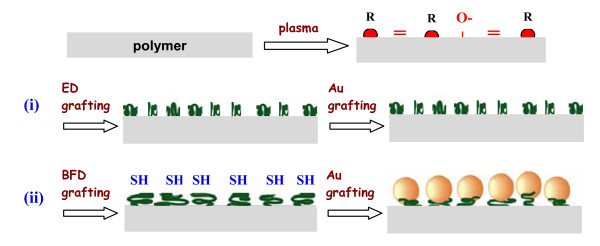
**Scheme of the plasma treatment of PET, grafting of modified PET**. By (i) ethanedithiol (ED) and (ii) biphenyldithiol (BFD) + then grafted by gold nanoparticles.

The progress of the present experiment and the main results of this work are schematically summarized in Figure 6. It was shown that the plasma treatment results in degradation of polymer chain and creation of free radicals, double bonds and excessive oxygen groups on the PET surface. The "flexible" molecule of 1, 2-ethanedithiol is bonded to the surface radicals probably by both of -SH groups in contrast to the "rigid" molecule of 4, 4'-biphenyldithiol, where one of -SH group remains "free" for the consecutive chemical reaction with the gold nanoparticle. The gold nanoparticles are grafted on the PET surface only in the case the pretreatment with 4, 4'-biphenyldithiol.

The presence of the -SH groups, as same as the gold nanoparticles on the grafted polymers was proved by XPS, FTIR, electrokinetic analysis and AFM methods. The gold nanoparticles are distributed relatively homogenously over the PET surface; this finding may be of importance for the future application of gold-polymer structures in tissue engineering and electronics.

## Competing interests

The authors declare that they have no competing interests.

## Authors' contributions

VŠ provided the idea, conceived of the study and designed and drafted the paper. ZK carried out the electrokinetic analysis. OK participated in FTIR measurements and its evaluation. JS carried out the AFM measurements and participated in its evaluation. AR modified PET surface and grafted it with dithiols. PŘ and KZ carried out the Au nanoparticle synthesis. All authors read and approved the final manuscript.
